# Microbiome-mediated regulation of chemoradiotherapy response

**DOI:** 10.3389/fonc.2025.1659467

**Published:** 2025-10-01

**Authors:** Lan Zhou, Benhua Li, Juan Ren, Shoujin Wang, Jun Wang

**Affiliations:** ^1^ Department of Clinical Laboratory, Affiliated Hospital of Panzhihua University, Panzhihua, China; ^2^ Department of Clinical Laboratory, The Second People ‘ s Hospital of Liangshan Yi Autonomous Prefecture, Xichang, China

**Keywords:** gut microbiota, chemoradiotherapy, immune checkpoint inhibitors, probiotics, microbial metabolites, dysbiosis

## Abstract

The gut microbiota critically influences patient responses to chemoradiotherapy through bidirectional interactions with host physiology, modulating both therapeutic efficacy and toxicity. Radiotherapy and chemotherapy disrupt microbial homeostasis, exacerbating intestinal damage, systemic inflammation, and immune dysfunction, while specific commensals and metabolites enhance treatment response via metabolic reprogramming, DNA repair regulation, and immune activation. Key mechanisms include microbiota-mediated TLR/NF-κB signaling, SCFA-dependent epigenetic modifications, and microbial enhancement of immune checkpoint inhibitors. Clinical interventions such as probiotics, fecal microbiota transplantation, and targeted antibiotics demonstrate potential to mitigate toxicity and overcome resistance. This review summarizes emerging evidence on how microbial dysbiosis induced by radiotherapy and chemotherapy exacerbates intestinal damage, systemic inflammation, and immune dysfunction, while specific commensals and metabolites enhance chemoradiotherapy response via metabolic reprogramming, DNA repair modulation, and immune activation. These findings underscore the gut microbiota as a critical determinant of chemoradiotherapy precision, offering actionable targets for microbiome-guided therapeutic optimization.

## 1 Introduction

The gut microbiota profoundly influences host responses to chemoradiotherapy by modulating immune pathways, DNA repair, and metabolic activity (1, 2). Anticancer treatments often induce dysbiosis—marked by reduced *Bifidobacterium* and *Lactobacillus* and expansion of pathogens like enterotoxigenic *Bacteroides fragilis*—which exacerbates mucosal damage, inflammation, and therapeutic toxicity (3). In contrast, specific microbes enhance treatment efficacy (4). For instance, *Bacteroides vulgatus* promotes DNA repair in tumor cells, impacting chemoradiation outcomes, while *Lactobacillus rhamnosus* improves radioprotection via the TLR2–ROS–Nrf2 axis (5–7). Microbial metabolites such as short-chain fatty acids (SCFAs) regulate histone deacetylase activity and enhance immune activation, thereby potentiating antitumor responses (8, 9).

Mechanistically, microbial signaling through TLR/NF-κB pathways, epigenetic modifications, and metabolic reprogramming affects both tumor biology and treatment response (10). Certain *Gammaproteobacteria* expressing cytidine deaminase inactivate gemcitabine, inducing resistance (11), while *Lactobacillus acidophilus* enhances oxaliplatin efficacy by stimulating macrophage ROS production (12). Furthermore, *Bifidobacterium* species increase CD8^+^ T cell infiltration and synergize with PD-L1 inhibitors, whereas *Bacteroides fragilis* supports CTLA-4 blockade via Th1 polarization (13, 14). Interventions such as probiotics, fecal microbiota transplantation (FMT), and targeted antibiotics offer potential to restore microbial balance, mitigate toxicity, and enhance therapeutic precision (15). This review summarizes the microbiome–therapy interactions and their translational implications in oncology, with a focus on molecular pathways governed by microbial metabolites, to inform microbiome-targeted strategies for optimizing cancer treatment outcomes.

## 2 Gut microbiota in tumor progression

Advanced culture-independent techniques, including metagenomic and 16S rRNA sequencing, facilitate comprehensive microbial characterization (16). Germ-free animal and FMT studies underscore the microbiota’s dual roles: symbiotic strains restrain tumorigenesis, whereas dysbiosis fosters oncogenic progression (17). Reduced bacterial diversity heightens breast cancer susceptibility, with antibiotic exposure disrupting estrogen homeostasis and amplifying HER-2/neu-driven tumorigenesis (18). Clinically, elevated fecal *Blautia* abundance associates with advanced histopathological stages (19). Probiotic administration mitigates Th17 responses, enhances regulatory T cell (Treg) activity, and inhibits hepatocellular carcinoma via IL-6 and TNF-α suppression (20, 21). The SCFAs butyrate mediates antitumor activity by inhibiting histone deacetylases (HDACs) and stimulating GPR109A signaling (22, 23). Integrated multi-omics, especially metabolomics, reshape understanding of microbiota–cancer interactions (24). Metatranscriptomics reveals stress-responsive microbial genes governing DNA repair and immunometabolic adaptation during therapy (25), while metabolomics identifies bioactive metabolites influencing epithelial barrier integrity (26). Together, these approaches provide high-resolution insight into host–microbiome–therapy dynamics, advancing predictive biomarker discovery and precision-targeted interventions.

Microbial ligands such as lipopolysaccharides (LPS), peptidoglycan, and bacterial CpG-DNA serve as MAMPs that engage PRRs like Toll-like receptors (TLRs) and nucleotide-binding oligomerization domain (NOD) proteins (27, 28). For instance, LPS derived from Gram-negative bacteria binds TLR4 on dendritic cells, triggering MyD88-dependent signaling cascades that activate NF-κB, leading to transcription of pro-inflammatory cytokines such as IL-1β, IL-6, and TNF-α (29, 30). Simultaneously, IL-6 and IL-23 promote STAT3 phosphorylation in T cells, favoring differentiation into Th17 cells over Tregs (31). In contrast, certain symbionts like *Bacteroides fragilis* produce polysaccharide A, which activates TLR2 and promotes IL-10–secreting regulatory T cells, suggesting that microbial pathway engagement can have dual outcomes depending on context and composition (32). Disrupted crosstalk between microbial components and pattern recognition receptors (PRRs) is a pivotal driver of malignant transformation (33). Microbial-associated molecular patterns (MAMPs) engage Toll-like receptors (TLRs), activating Ca²^+^/calcineurin-NFAT signaling to stimulate cellular proliferation (34). Aberrant TLR4 expression promotes oncogenesis in pulmonary, hepatic, and colorectal cancers, whereas TLR2 upregulation contributes to gastric carcinogenesis (35–38). Cooperative NF-κB/STAT3 signaling suppresses apoptosis via NF-κB while promoting angiogenesis and immune evasion through STAT3-dependent VEGF and PD-L1 expression (39–41), underscoring the microbiota’s dual regulatory role in tumor–immune modulation.

## 3 Interplay between intestinal microbiota and radiotherapy

### 3.1 Gut microbiota in radiotherapy response

Radiotherapy disrupts gut microbiota by damaging epithelial DNA, reducing stem cells, and impairing mucus barriers, leading to decreased diversity (*Bacteroidetes*/*Firmicutes* imbalance), pathogenic overgrowth (*Enterobacteriaceae*), and loss of beneficial taxa (*Bifidobacterium*) (42). These alterations exacerbate radiation-induced toxicity. Specifically, diminished SCFA production impairs metabolism, while immune dysfunction emerges through elevated IL-1β/TNF-α and a Th17/Treg imbalance (43–48). Moreover, microbial composition has predictive value. Patients with richer microbiota profiles respond better to lower radiation doses. In contrast, individuals with dysbiotic microbiota often require intensified regimens, highlighting the microbiome’s potential as a biomarker for personalized dosing (49–52). Clinical evidence further supports this. Fecal microbial gene signatures correlate with the severity of radiation-induced intestinal injury, enabling real-time stratification of patients undergoing pelvic irradiation (53). Notably, pelvic radiotherapy elevates *Blautia* levels, which associates with compromised gut barrier function, electrolyte dysregulation, and diarrhea. Interventions such as probiotic supplementation (*Lactobacillus/Bifidobacterium*) mitigate enteritis by reinforcing tight junctions and suppressing TLR4-NF-κB signaling (54, 55). FMT has also demonstrated efficacy in reestablishing microbial equilibrium, promoting mucosal healing, and overcoming radioresistance (56). Mechanistically, specific strains such as *Lactobacillus rhamnosus* enhance radiotherapy efficacy by activating the TLR2-ROS-Nrf2 pathway. This augments antioxidant responses and facilitates CD8^+^ T cell recruitment to the tumor site (48). Together, these insights elucidate the molecular mechanisms governing microbiota-radiotherapy interactions, providing a foundation for microbiome-informed precision radiation oncology.

### 3.2 Microbial dysbiosis and barrier dysfunction in radiotherapy

Radiation therapy profoundly perturbs gastrointestinal microbial homeostasis through direct DNA damage to crypt stem cells induced by ionizing radiation. This triggers p53-mediated apoptosis, compromises barrier integrity (20), and initiates a pathological cascade. Reduced Paneth cell secretion of antimicrobial peptides weakens mucosal defense, allowing expansion of opportunistic taxa (*Bacteroidaceae*, *Lactobacillaceae*) while depleting *Clostridiaceae*, a key source of SCFAs (57). The ensuing dysbiosis activates dendritic cells via TLR4–NF-κB signaling, driving excessive release of pro-inflammatory cytokines (IL-1β, IL-6, TNF-α) and reinforcing a cycle of radiation-induced epithelial injury, microbial imbalance, and inflammation (58). In Wistar rats, whole-body irradiation elicited dose-dependent microbiota shifts, with increased *Bacteroidaceae* and *Lactobacillaceae* 16S rRNA alongside *Clostridiaceae* depletion, suggesting this signature as a potential biomarker of radiation exposure (59). Overgrowth of pathobionts further compromises epithelial permeability, enabling bacterial translocation and systemic inflammation that underlie post-radiotherapy complications such as diarrhea and electrolyte imbalance (58). Notably, dysbiosis and barrier dysfunction act bidirectionally: reduced SCFAs impair epithelial regeneration, while persistent injury exacerbates microbial disruption, establishing a vicious cycle of sustained tissue damage (54). These findings highlight microbial modulation as a promising therapeutic approach for radiation enteritis, with strategies targeting *Clostridiaceae* restoration and TLR4-NF-κB pathway inhibition emerging as critical interventions (59).

### 3.3 Radiation-triggered systemic antitumor immunity shaped by gut microbiota

The abscopal phenomenon in radiotherapy depends on radiation-induced antigen exposure, cross-priming by dendritic cells, and subsequent systemic T-cell activation (60). In TSA breast carcinoma and MCA38 colon adenocarcinoma models, the combined fractionated radiation and CTLA-4 inhibition synergistically enhance systemic antitumor immunity, emphasizing the critical role of adaptive immune responses (61). The intestinal microbiome significantly influences immune function through gut-associated lymphoid tissue (GALT). Studies in axenic mice reveal underdeveloped Peyer’s patches containing diminished CD4^+^ T lymphocyte populations and decreased IgA-secreting B cells, resulting in compromised tumor surveillance (50, 51, 62). In contrast, colonization with segmented filamentous bacteria (SFB) potentiates antigen presentation through TLR4 stimulation and modified bile acid metabolism (63). Secondary bile acids, such as deoxycholic acid and lithocholic acid, act through the farnesoid X receptor (FXR) and G-protein-coupled bile acid receptor 1 (TGR5) to modulate intestinal immunity and regulate inflammation, impacting systemic T cell activation and radiosensitivity. Microbial metabolites, particularly short-chain fatty acids (SCFAs), mediate the conversion of regulatory T cells into tumoricidal CD8^+^ T lymphocytes, amplifying intratumoral immune cell infiltration following radiation treatment (50, 51). Inosine, a purine nucleoside produced by *Bifidobacterium pseudolongum*, enhances T cell-mediated killing via activation of A_2_A receptors, particularly under conditions of low glucose availability within the tumor microenvironment, thereby synergizing with radiotherapy. Clinical observations demonstrate significant associations between microbial community richness and abscopal response rates, with specific metabolites such as indole-3-propionic acid augmenting CD8^+^ T cell cytotoxicity through aryl hydrocarbon receptor (AhR)-dependent pathways (64).

### 3.4 Engineering gut microbes for radiation benefits

**Figure 1:**
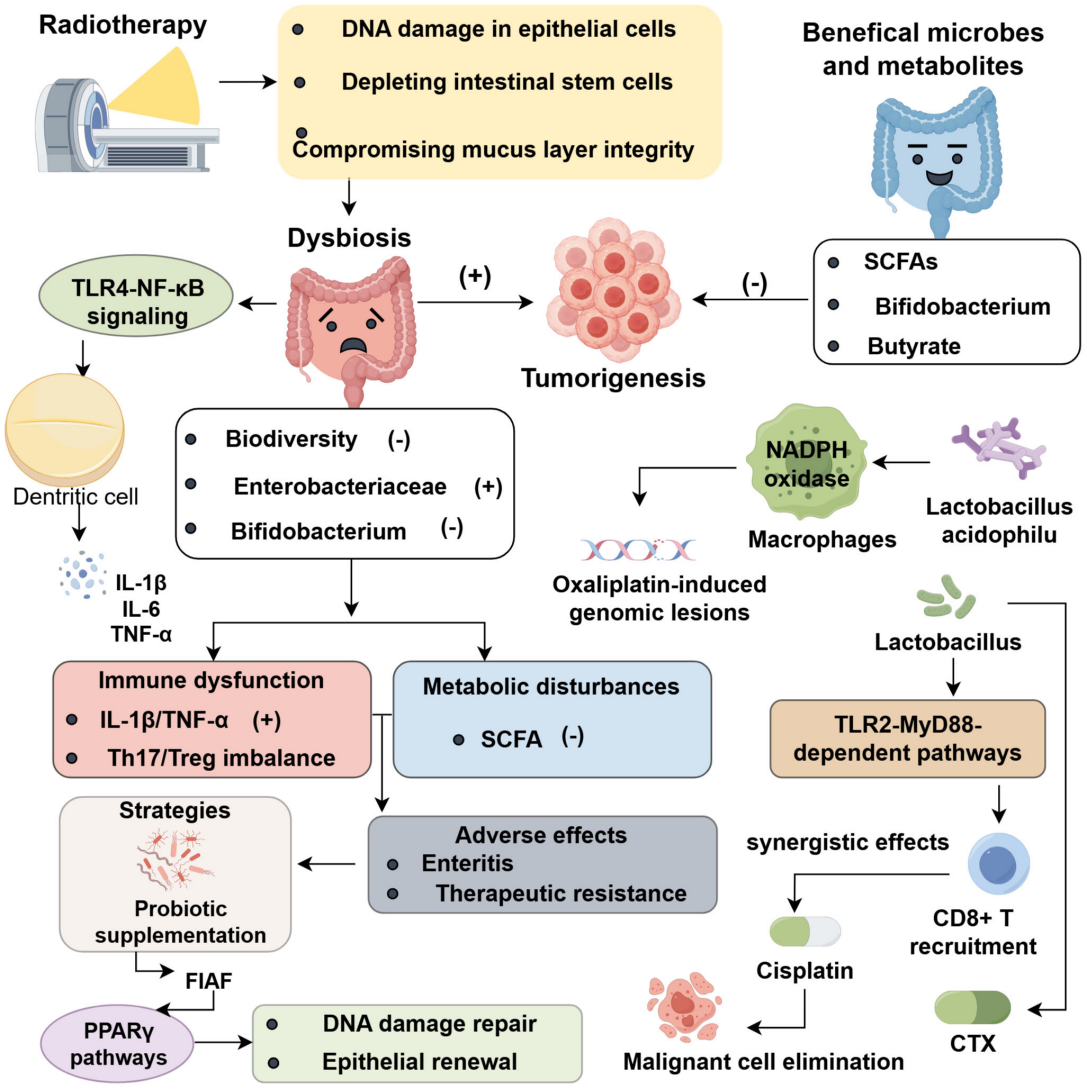
Roles of Gut Microbiota in Chemoradiotherapy Response.

The intestinal microbiota critically shapes radiation therapy outcomes by reprogramming metabolic networks and fine-tuning immune responses. Targeted interventions like probiotics, prebiotics, and fecal microbiota transplantation restore microbial balance, thereby augmenting tumor control while mitigating adverse effects (65). At the molecular level, fasting-induced adipose factor (FIAF), an epithelial protein regulated by microbes, activates PPARγ signaling to enhance DNA repair in the intestinal epithelium, with radioprotective effects observed in microbiota-depleted animal models (66). Specific microbial populations demonstrate distinct protective capacities: short-chain fatty acid-producing species (*Enterococcus faecalis*, *Clostridium perfringens*) attenuate radiation-induced oxidative damage through FIAF induction, whereas *Bacteroides* and *Escherichia coli* facilitate epithelial renewal via PPARγ-mediated mitochondrial proliferation (67, 68). Clinically, probiotic supplementation with *Streptococcus*, *Lactobacillus*, and *Bifidobacterium* augments FIAF expression through histone acetylation, synergizing with radiation to promote malignant cell apoptosis while reducing gastrointestinal toxicity (69, 70). The antibiotic vancomycin, with selective activity against Gram-positive bacteria, reshapes host–microbiome–immune dynamics to potentiate radiation-mediated tumor control. In B16-OVA melanoma and TC-1 carcinoma models, vancomycin reduces *Clostridiales* abundance, enhances MHC class I expression on dendritic cells, and augments IFN-γ-driven tumor clearance while modulating PD-L1 and CTLA-4 checkpoint pathways (63). Antibiotic-induced dysbiosis further activates TLR2–MyD88 signaling, reprogramming Tregs into Th1 cells within gut-associated lymphoid tissues, which enhances antigen-presenting cell migration to tumor-draining lymph nodes and establishes durable systemic immunity (71, 72). Microbiota also influence local Treg dynamics during radiotherapy: Bacteroides-derived tryptophan catabolites stabilize Tregs via AhR activation, whereas microbial disruption impairs radiation-induced immunogenic cell death (73) (**Figure 1**).

## 4 Gut microbiota in chemotherapy responses

### 4.1 Intestinal microbiota in platinum drug efficacy

Platinum-derived chemotherapeutics exert cytotoxicity by inducing DNA crosslinks, mitochondrial dysfunction, and oxidative injury, with reactive oxygen species (ROS) as central mediators. Emerging evidence reveals that the gut microbiota potentiates platinum efficacy through ROS regulation. *Lactobacillus acidophilus* enhances oxaliplatin-induced DNA lesions by activating macrophage NADPH oxidase while impairing non-homologous end joining repair (74). Conversely, antibiotic-driven microbial depletion attenuates oxidative stress and weakens drug response (58). Additionally, *Lactobacillus* promotes CD8^+^ T cell infiltration into tumors via TLR2–MyD88 signaling, synergizing with cisplatin to strengthen cytotoxicity and remodel immunoregulatory niches, thereby extending survival in murine lung cancer models (75). Clinically, antibiotic-associated microbial diversity loss correlates with diminished cisplatin efficacy, whereas probiotic supplementation with *L. acidophilus* restores responsiveness by reactivating TNF-α/IL-6–mediated inflammatory cascades. This recovery involves microbial short-chain fatty acids, notably butyrate, which epigenetically upregulate apoptotic genes (BAX, CASP3) through chromatin remodeling (59, 75).

### 4.2 Microbiota-driven modulation of cyclophosphamide efficacy

The alkylating agent cyclophosphamide (CTX) mediates antitumor efficacy through immunogenic cell death pathways that critically depend on microbial modulation of host immunity. CTX induces marked remodeling of the intestinal microbiota, characterized by reduced *Bacteroidetes* and enrichment of *Firmicutes*, including *Bacteroides acidifaciens* and *Alistipes*, which correlate with improved therapeutic response (76). CTX promotes translocation of Gram-positive bacterial components (*Lactobacillus* spp.) into secondary lymphoid tissues, where TLR4-dependent dendritic cell activation drives Th17 polarization and strengthens memory Th1 responses, leading to increased intratumoral IFN-γ–producing cytotoxic T cells (52). In axenic or vancomycin-treated mice, this immune axis is disrupted, while microbial reconstitution restores CTX efficacy, underscoring the indispensable role of commensals in therapy responsiveness (77). Additionally, CTX-driven microbial shifts suppress indoleamine 2,3-dioxygenase (IDO) activity, lowering kynurenine levels and diminishing Treg-mediated immunosuppression (52, 76), revealing potential microbial targets for enhancing chemotherapy response. The efficacy of gemcitabine, a nucleoside analog, is undermined not only by tumor-intrinsic resistance but also by microbial enzymatic degradation. *Gammaproteobacteria*, including Escherichia coli and *Pseudomonas aeruginosa*, secrete cytidine deaminase (CDD), which converts gemcitabine into its inactive metabolite 2′, 2′-difluorodeoxyuridine, a phenomenon especially prominent in pancreatic tumor microenvironments (78). Crucially, the bacterial CDD long isoform drives this inactivation: coculture experiments show that *Mycoplasma hyorhinis* strains expressing CDD confer complete resistance even at gemcitabine levels tenfold higher than therapeutic doses. In contrast, targeted depletion of intratumoral bacteria or pharmacological inhibition of CDD restores chemosensitivity (78). The identification of these mechanisms supports the development of microbiome-informed precision approaches to overcome chemoresistance.

### 4.3 Microbiome in irinotecan-induced gastrointestinal toxicity

The gastrointestinal toxicity of irinotecan arises from enterohepatic cycling and microbial enzymatic reactivation. After hepatic carboxylesterase-mediated conversion to the active metabolite SN-38, subsequent glucuronidation produces the inactive conjugate SN-38-G, which is secreted into the intestinal lumen (78). Commensal bacteria, particularly Enterobacteriaceae and *Clostridium* species, expressing β-glucuronidase, hydrolyze SN-38-G back to its active form, thereby disrupting epithelial integrity and provoking severe diarrhea (48). At the molecular level, β-glucuronidase-producing strains, exemplified by *Escherichia coli* K-12, amplify intestinal inflammation via TLR4–MAPK signaling, with their abundance correlating with clinical diarrhea severity. Interventions that inhibit β-glucuronidase or reshape microbial communities through probiotics (*Lactobacillus*) significantly reduce luminal SN-38 levels, alleviating chemotherapy-induced diarrhea (79). Microbiome profiling reveals that the relative abundance of *Firmicutes* versus *Bacteroidetes*, along with overall microbial diversity, serves as predictive biomarkers for irinotecan-related enterotoxicity risk. Patients exhibiting diminished microbial richness show increased susceptibility to severe gastrointestinal complications, highlighting the potential of microbiota characterization for optimizing individualized treatment protocols to minimize adverse effects (**Supplementary Table S1**).

### 4.4 TLR9 agonists and microbiota in antitumor response

The immunostimulatory potential of TLR9 is mediated by recognition of bacterial CpG motifs, whereby synthetic CpG oligodeoxynucleotides (CpG-ODNs) engage TLR9 on antigen-presenting cells, triggering tumor necrosis factor (TNF) release, immunogenic apoptosis, and hemorrhagic tumor destruction (80). Antibiotic-induced depletion of Gram-negative bacteria reduces LPS availability, suppressing TNF, IL-1α, and IL-12β production by tumor-infiltrating myeloid cells and abolishing CpG-ODN efficacy in colorectal carcinoma. Restoration of TLR4 signaling through exogenous LPS reactivates MyD88/TRIF-dependent cascades, reversing antibiotic-mediated immunosuppression and confirming microbial regulation of antitumor responses via TLR4 pathways (49). At the molecular level, the gut commensal Alistipes shahii potentiates CpG-ODN activity through sphingolipid-mediated NLRP3 inflammasome activation in dendritic cells, driving IL-1β-mediated lymphocyte recruitment and tumor microenvironment modification (81, 82). Together, these findings underscore microbial–TLR crosstalk as a critical determinant of immunotherapeutic success, supporting microbiome-informed strategies that integrate TLR agonists with targeted microbial modulation.

## 5 Microbiome regulation of immune checkpoint inhibitor efficacy

The therapeutic activity of immune checkpoint inhibitors (ICIs) targeting CTLA-4 and PD-1/PD-L1 pathways is fundamentally regulated by intestinal microbiome-mediated immune modulation (83–85). CTLA-4 blockade induces Treg depletion while promoting cytotoxic T lymphocyte (CTL) accumulation, whereas PD-L1 inhibition reverses T cell dysfunction by downregulating inhibitory receptors (TIM-3, LAG-3) (86). Specific bacterial species demonstrate immunostimulatory effects: *Bacteroides thetaiotaomicron* and *B. fragilis* potentiate CTLA-4 inhibitor responses through dendritic cell activation via TLR4/MyD88-dependent mechanisms, elevating IL-12 production and Th1 polarization (61). Likewise, *Bifidobacterium* species potentiate PD-L1 blockade by modulating indole-3-propionic acid metabolism, thereby improving mitochondrial oxidative fitness in CD8^+^ T cells (50). Clinically, antibiotic-induced disruption of *B. fragilis* impairs CTLA-4 inhibitor responses, an effect reversible by bacterial reconstitution (51). Besides, microbial community diversity influences tumor progression kinetics, with FMT or *Bifidobacterium* supplementation restoring dendritic cell cross-priming and CD8^+^ infiltration, yielding antitumor effects comparable to PD-L1 blockade and complete responses in combination. These findings delineate a sophisticated microbiome-immune-metabolism network that critically determines ICI responsiveness, offering new avenues for microbial-based therapeutic interventions.

## 6 Microbiota-mediated protection in chemotherapy and radiotherapy

Beneficial microorganisms exert therapeutic effects against chemotherapy-induced intestinal injury by reprogramming host–microbiome interactions, primarily through suppression of ROS and modulation of pattern recognition receptor pathways (87, 88). Probiotic consortia containing *Lactobacillus acidophilus* and *Bifidobacterium* spp. reduce cisplatin-associated mucosal inflammation and preserve epithelial barrier integrity in preclinical colorectal cancer models via inhibition of NADPH oxidase–driven oxidative stress (89). The pathophysiology of methotrexate-related intestinal damage involves TLR4-MyD88/TRIF signaling activation, whereby cytotoxic agents induce Gram-negative bacterial cell wall disintegration and subsequent LPS liberation, initiating pro-inflammatory TLR4-mediated responses. Probiotic administration counteracts this process through competitive exclusion of pathogenic organisms, consequently limiting LPS-mediated enterocyte programmed cell death and junctional protein breakdown (43). Notably, certain isolates such as *Lactobacillus rhamnosus* GG secrete extracellular polysaccharides that regulate NLRP6 inflammasome function (90), establishing a protective microbiota–gut–liver axis and offering innovative avenues for mitigating chemotherapy-related complications.

Radiotherapy-induced cytotoxicity in intestinal crypt cells disrupts mucosal integrity, driving microbial imbalance (*Bacteroidetes*/*Firmicutes* ratio) and increased gut permeability, which underlie sequelae such as radiation enteritis and oral mucositis (79, 91). Probiotic administration with *Lactobacillus acidophilus*, *Bifidobacterium*, and *Lactobacillus casei* mitigates oxidative stress via Nrf2–ARE activation, significantly reducing severe (grade ≥3) enteritis while preserving barrier integrity through maintenance of tight junction complexes (80). At the molecular level, these commensals modulate host immunity by engaging TLR2/NF-κB signaling with microbial-derived SCFAs, thereby suppressing radiation-induced cytokine cascades (IL-6, TNF-α). Clinical studies in head and neck cancer further demonstrate that Lactobacillus-based interventions reduce radiation-associated oral mucositis by 42.7%, mediated through enhanced salivary EGF secretion and upregulated MUC2 glycoprotein synthesis, ultimately improving adherence to therapy (58). Collectively, these findings define the microbiota–immune interface as a critical determinant of radiotherapy tolerance and provide a translational rationale for microbiota-targeted strategies in mitigating treatment-related complications.

## 7 Conclusion

Accumulating evidence identifies the gut microbiota as a key regulator of chemoradiotherapy efficacy through modulation of immune activation, epithelial repair, and DNA damage responses. Commensal species such as Bifidobacterium and Lactobacillus enhance antitumor immunity by increasing CD8^+^ T cell infiltration and promoting the effectiveness of immune checkpoint blockade. In parallel, microbial metabolites including SCFAs, bile acids, inosine, and polyamines influence histone acetylation, redox balance, and cytotoxic T cell function, offering new avenues for therapeutic enhancement. Microbial enzymatic activity can also shape drug metabolism and resistance, exemplified by gemcitabine inactivation via bacterial cytidine deaminase.

Nevertheless, substantial barriers hinder clinical translation. Interindividual variability in microbial composition driven by genetics, diet, and environment poses a challenge for standardizing interventions such as probiotics or fecal microbiota transplantation. Safety concerns must also be considered, including the risk of bacteremia, off-target immune modulation, and unintended dysbiosis in vulnerable patients. Moreover, a lack of validated microbial biomarkers and prospective clinical trials limits the predictive utility of microbiome-based strategies. Future research should focus on personalized microbial profiling, rational microbial consortia design, and rigorous mechanistic validation to enable safe and effective microbiota-informed oncology care.
